# Development and validation of a two-dimensional liquid chromatography method for therapeutic drug monitoring of pyrotinib in human plasma

**DOI:** 10.3389/fphar.2025.1704594

**Published:** 2025-11-21

**Authors:** Chunxia Gao, Yuzhi Cao, Mengna An, Zhihui Li, Chao Meng, Jingjing Lan, Haisheng You, Siying Chen

**Affiliations:** 1 Department of Pharmacy, The First Affiliated Hospital of Xi’an Jiaotong University, Xi’an, Shaanxi, China; 2 Department of Clinical Pharmacy, Tongchuan People’s Hospital, Tongchuan, Shaanxi, China; 3 Department of Pharmacy, Xi’an Ninth Hospital, Xi’an, Shaanxi, China

**Keywords:** pyrotinib, two-dimensional liquid chromatography, plasma drug concentration, therapeutic drug monitoring, validation, HER2-positive breast cancer

## Abstract

**Objective:**

To develop and validate a method for therapeutic drug monitoring (TDM) of pyrotinib in human plasma using two-dimensional liquid chromatography (2D-LC) system.

**Method:**

The plasma samples were pretreated with acetonitrile for protein precipitation. The mobile phase consisted of two parts: a first-dimensional mobile phase (methanol, acetonitrile, and 65 mmol/L ammonium phosphate in a ratio of 1:3:3, V/V/V) and a second-dimensional mobile phase (acetonitrile, isopropanol, and 10 mmol/L ammonium phosphate in a ratio of 16:7:1, V/V/V). The analysis cycle time was completed within 9.50 min. The method was validated for linearity, recovery, precision, accuracy, and stability.

**Results:**

Pyrotinib demonstrated excellent linearity within the range of 10.10–810.40 ng/mL with regression equation y = 556.4044× + 462.40 (*R* ^2^ = 0.9995). The relative recovery rate of plasma samples was stable and reproducible, ranging from 96.82% to 100.12%. The intra-day and inter-day precisions were ≤5.30% and ≤3.80% for pyrotinib concentrations, respectively. Stability tests confirmed that pyrotinib in plasma remained stable under the following conditions: room temperature for 8 h, 4 °C for 48 h, −20 °C for 3 weeks, three freeze-thaw cycles. This method was validated in twenty patients with advanced HER2-positive breast cancer (dose range: 240–400 mg/day) The trough and peak plasma concentrations of pyrotinib ranged from 17.75–92.56 ng/mL and 51.17–232.94 ng/mL, respectively, which demonstrated significant pharmacokinetic heterogeneity.

**Conclusion:**

The developed 2D-LC analytical method not only demonstrates good precision, accuracy, recovery, and stability, but also is simple, rapid, feasible, and practical for TDM. It can be used for the concentration monitoring of pyrotinib in clinic, providing more scientific evidence for clinical practice.

## 1 Introduction

Breast cancer is the leading cause of cancer-related deaths among women worldwide (Lei et al., 2022). The prevalence of breast cancer continues to rise annually, with projections indicating 4.4 million new cases by 2070 (Soerjomataram and Bray, 2021; Harbeck and Gnant, 2017). Human epidermal growth factor receptor 2 (HER2) positive breast cancer is characterized by high recurrence rates and poor prognosis and occurs in 15%–20% (Loibl and Gianni, 2017). Current treatments for HER2-positive breast cancer include tyrosine kinase inhibitors (TKIs), monoclonal antibodies, and antibody-drug conjugates (Shahid et al., 2015).

Pyrotinib, an originally developed irreversible small-molecule TKI originated by Jiangsu Hengrui Medicine, was approved by National Medical Products Administration in China in 2018 for the treatment of HER2-positive recurrent or metastatic breast cancer. It was licensed to Korean HLB-LS Company in 2020 and to Dr. Reddy’s, a publicly listed Indian company in 2023, indicating potential for future clinical use (https://www.hengrui.com/international/index.html). It also indicated that pyrotinib was combined with trastuzumab and docetaxel to cure HER2-positive advanced breast cancer as a first-line treatment option (Xu et al., 2021). Compared to traditional antibody-based therapies like trastuzumab, pyrotinib comprehensively inhibits downstream signaling pathways of both homo-and heterodimers within the HER family. Pyrotinib may still be effective for patients resistant to trastuzumab. Its mechanism of action differs from that of trastuzumab. Pyrotinib can irreversibly bind to the ATP-binding site, thereby blocking the HER2 signaling pathway and overcoming trastuzumab resistance (Song et al., 2020). Additionally, its low molecular weight and structural flexibility enhance blood-brain barrier penetration, demonstrating therapeutic efficacy against breast cancer brain metastases (Luo et al., 2023). Despite its significant clinical benefits in monotherapy or combination regimens, which prolong overall survival, severe adverse reactions (e.g., grade ≥3 events) and long-term drug resistance remain critical clinical concerns. In the PHILA trial, although no pyrotinib-related deaths were reported, 90% of patients experienced grade ≥3 treatment-related adverse events (Zhang et al., 2024). Monotherapy with pyrotinib resulted in a 44.7% incidence of diarrhea (Li et al., 2019), with severe cases being the primary cause of treatment discontinuation and poor adherence. Other common adverse effects included hand-foot syndrome, neutropenia, and hepatic dysfunction (Hu et al., 2020; Zheng et al., 2021; Tian et al., 2020; Ren et al., 2023). In order to enhance the therapeutic efficacy of pyrotinib and reduce the incidence of its adverse reactions, it is necessary to conduct therapeutic drug monitoring (TDM) for pyrotinib.

Early studies primarily employed radio-liquid chromatography (Radio-LC) and ultra-performance liquid chromatography (UPLC) to investigate pyrotinib pharmacokinetic profiles (Meng et al., 2019; Zhu et al., 2016). More recently, ultra-performance liquid chromatography–tandem mass spectrometry (UPLC-MS/MS) has become the method of choice for population pharmacokinetic studies because of its superior stability, sensitivity and throughput (Zhu et al., 2024). Nevertheless, UPLC-MS/MS requires expensive instrumentation, specialized maintenance and strict environmental controls. As an alternative method, two-dimensional liquid chromatography (2D-LC) provides comparable quantitative performance while offering simpler operation, lower running costs and minimal maintenance, making it particularly attractive for routine TDM. Consequently, an increasing number of hospitals have adopted 2D-LC platforms for plasma concentration measurement of various drugs (Liu et al., 2022; Kong et al., 2022; Zhao et al., 2021; Yan et al., 2024; He et al., 2024; Xie et al., 2024). The present study therefore aimed to develop and fully validate a 2D-LC method for pyrotinib quantification in human plasma to support TDM and guide individualized dosing decisions.

## 2 Materials and methods

### 2.1 Drugs and reagents

Pyrotinib maleate reference standard (Batch No.: 420062-201901, purity: 98.70%) was purchased from the National Institutes for Food and Drug Control (Beijing, China). Acetonitrile, methanol, 65 mmol/L ammonium phosphate, isopropanol and 10 mmol/L ammonium phosphate were all purchased from Hunan Dimat Instruments Co., Ltd., Ultrapure water was produced using a Millipore Direct-Q5 Water Purification System (Merck, America). Blank human plasma and patient plasma samples were provided by the Clinical Laboratory of the hospital (The First Affiliated Hospital of Xi’an Jiaotong University, China). All patients signed informed consent. This study protocol was reviewed and approved by the Ethics Committee of the First Affiliated Hospital of Xi’an Jiaotong University, Approval No (XJTU1AF2024LSYY-140).

### 2.2 Instrumentation and principles

The 2D-LC system (model FLC2420) comprised an FLC 2D-LC coupling unit (Hunan Demeter Instruments Co., Ltd., China) and Shimadzu LC-20AT liquid chromatography modules. The system was operated by the LabSolutions workstation (Shimadzu, Japan). Additional equipment included a GH-202 electronic analytical balance (A&D Company, Ltd., Japan) and an XW-80A vortex mixer (Shanghai Qite Analytical Instruments Co., Ltd., China).

The 2D-LC system integrates functional modules such as the first-dimensional chromatographic pump, second-dimensional chromatographic pump, detector, and autosampler. The system is configured to operate as two independent subsystems: the first-dimensional liquid chromatography system (LC1) and the second-dimensional liquid chromatography system (LC2), based on predefined operational modes. The first-dimensional column (LC1 column) primarily conducts online sample enrichment and preliminary separation, whereas the second-dimensional column (LC2 column) accomplishes refined separation and detection. These two subsystems are interconnected via an intelligent flow path control system (TRS), which operates in heart-cutting mode, employing an intermediate column for the targeted transfer of analytes between dimensions.

### 2.3 Chromatographic conditions

**FIGURE 1:**
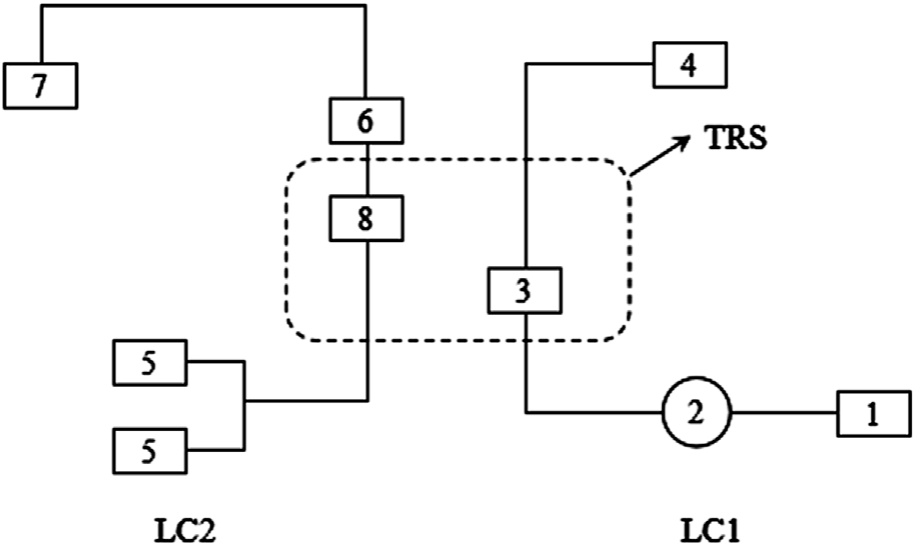
Working program of 2D-LC. LC1: First-dimensional liquid chromatography system; 1: first-dimensional liquid chromatography pump; 2: connecting autosampler; 3: first-dimensional liquid chromatography column; 4: waste liquid collection bottle; LC2: Second-dimensional liquid chromatography system; 5: second-dimensional liquid chromatography pump; 6: second-dimensional liquid chromatography column; 7: detector; 8: intermediate trap columns; TRS: trap column transfer system.

The first-dimension chromatographic column employs an Aston SX1 (3.5 × 25 mm, 5 µm) cation-exchange column, while the intermediate column utilizes an Aston SCB (4.6 × 10 mm, 3.5 µm) C18 column to resolve acids, reduce diffusion, refocus, and avoid excessive exchange of substances. The second-dimension column is an Aston SCB (4.6 × 125 mm, 5 µm) C18 column. The first-dimension mobile phase consisted of methanol, acetonitrile, and 65 mmol/L ammonium phosphate (in a ratio of 1:3:3, V/V/V, pH adjusted to 7.0 with triethylamine). The second-dimension mobile phase consisted of acetonitrile, isopropanol, and 10 mmol/L ammonium phosphate (in a ratio of 16:7:1, V/V/V), with a total pump flow rate of 1.2 mL/min. The detection wavelength is 356 nm. The column temperature is 40 °C, and the injection volume is 300 µL. The timed program corresponding to the workflow diagram is detailed in Table 1. The working principle of the 2D-LC system is shown in Figure 1.

**TABLE 1:** The timing program of 2D-LC.

Work order	Process 1	Process 2	Process 3
Time (min)	0–3 min	3–5 min	5–9.5 min
Working status	LC1 Chromatographic Column Work, Preliminary Sample Separation	LC1 Connected with Intermediate Column, Capture of Target Components in the Sample	LC2 Connected with Intermediate Column, Separation detection of Target Components in the Sample

2D-LC, two-dimensional liquid chromatography.

### 2.4 Solution preparation

20.50 mg of pyrotinib maleate reference standard was accurately weighed (purity: 98.70%). The mass of 14.47 mg pyrotinib was calculated based on the given purity. The sample was dissolved with methanol in a 25 mL volumetric flask and diluted to the mark, resulting in a stock solution of pyrotinib with a concentration of 0.5788 mg/mL. The stock solution was stored at −20 °C for later use. Table 2 is provided below.

**TABLE 2:** Preparation of pyrotinib standard solution.

Pyrotinib references standard	Stock solution	Working solution
Purity: 98.70% (mg)	20.50 (actual mass: 14.47)	—
Solvent	Methanol	Blank human plasma
Volume (mL)	25	Withdraw the corresponding volume
Stock solution conc (mg/mL)	0.5788	Nominal conc/QC
Storage conditions	−20 °C	−20 °C

### 2.5 Preparation of standards and quality control samples

The stock standard solutions of pyrotinib were prepared by diluting the stock solution with blank human plasma. The stock solutions were accurately diluted with blank human plasma to prepare six calibration standards (10.10, 50.60, 101.30, 202.60, 405.20, and 810.40 ng/mL). The standards were stored at −20 °C until use. The calibration range was established based on the pyrotinib phase I clinical trial (38.8–175 ng/mL, Ma et al., 2017) and published literature values (32.60–82.80 ng/mL, Zhao et al., 2021).

The quality controls (QC) solutions of pyrotinib were also prepared by dissolving the stock solution with blank human plasma. There are three concentration levels: low (45 ng/mL), medium (180 ng/mL), and high (720 ng/mL) concentrations. The prepared samples were subsequently aliquoted into QC vials and stored at −20 °C.

### 2.6 Plasma sample pretreatment

500 μL protein treatment reagent of acetonitrile was accurately pipetted into a microcentrifuge tube, then 200 μL plasma sample was added. The mixture was thoroughly vortex-mixed and subjected to high-speed centrifugation at 14,500 rpm for 8 min. The resulting supernatant was then transferred to an injection vial for subsequent analysis.

### 2.7 Method validation

The analytical method was validated according to the “9012 guidelines for validation of quantitative analytical methods for biological samples” from the Chinese Pharmacopoeia (2020 edition) (The State Pharmacopoeia Commission of P.R. China, 2020) (Ch.p, 2020) and the validation guidelines for biological sample analysis methods issued by the US Food and Drug Administration (FDA) (US Department of Health and Human Services, 2018) (FDA, 2018), the validation process encompassed selectivity, standard curve, lower limit of quantitation, accuracy and precision, extraction recovery, and stability.

QC samples of pyrotinib at low (45 ng/mL), medium (180 ng/mL), and high (720 ng/mL) concentrations were accurately pipetted and processed according to the “Plasma Sample Pretreatment” procedure, and then analyzed under the conditions described in “Chromatographic Conditions.” The samples were analyzed five times within a single day (intra-day) and across three independent batches (inter-day) to calculate precision and accuracy. The accuracy was represented by the percentage of the measured concentration of the target analyte to the nominal concentration, which should be within the range 85%–115% (80–120% for the LLOQ). The precision was represented by the relative standard deviation (RSD, %), which should be ≤15% (≤20% for the LLOQ).

QC samples of pyrotinib at low (45 ng/mL), medium (180 ng/mL), and high (720 ng/mL) concentrations (n = 5 for each level) were extracted and analyzed once to determine recovery efficiency, using the identical pretreatment and chromatographic conditions. The peak areas of these processed samples were compared to those of reference standard working solutions at corresponding concentrations, with five replicates per concentration. The extraction recovery should be within the range 85%–115% (80%–120% for the LLOQ), with a precision (RSD, %) of ≤15% (≤20% for the LLOQ).

Quality control (QC) samples at low (45 ng/mL), medium (180 ng/mL), and high (720 ng/mL) concentrations were subjected to various stability tests: Room temperature (RT) stability, where samples were stored at RT for 4, 8, and 24 h; Freeze-thaw stability, involving three freeze-thaw cycles; Short-term storage, with samples kept at 4 °C for 8, 24, and 48 h; and Long-term storage, where samples were frozen at −20 °C for 1, 2, and 3 weeks. All samples were processed using the identical pretreatment and chromatographic conditions. If the accuracy of the processed sample is within the range 85%–115%, with a precision (RSD %) of ≤15%, then the sample is considered stable.

### 2.8 Clinical application validation

This study enrolled twenty patients with advanced breast cancer who were receiving pyrotinib. Inclusion criteria: only patients with recurrent/metastatic breast cancer whose pathology reports explicitly documented HER2 (IHC 3+) or FISH amplification-positive status were enrolled. The applicability of the established method for detecting plasma concentrations of pyrotinib was verified through quantitative analysis of the peak and trough concentrations in the plasma of HER2-positive breast cancer patients receiving pyrotinib treatment.

### 2.9 Statistical methods

This study relied on basic statistics. We calculated Means ± SD and evaluated RSD with descriptive methods, then derived the regression equation through linear regression.

## 3 Results

### 3.1 Specificity and chromatograms

**FIGURE 2:**
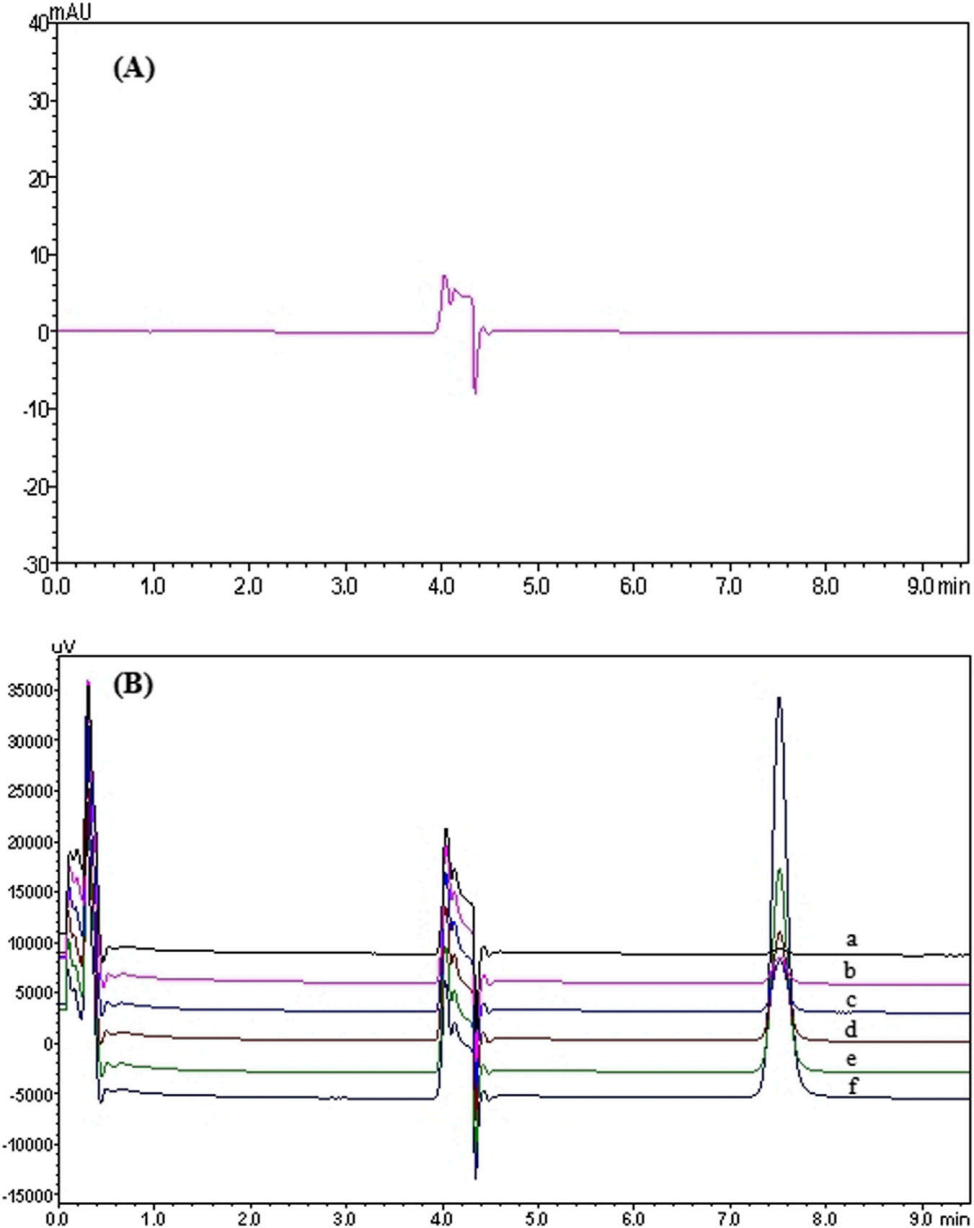
2D-LC chromatogram of Pyrotinib: Chromatograms of pyrotinib in blank plasma **(A)** and the standard curve (six concentrations were as follows: 10.10 (a), 50.60 (b), 101.30 (c), 202.60 (d), 405.20 (e), 810.40 (f) ng/mL) **(B)**.

**FIGURE 3:**
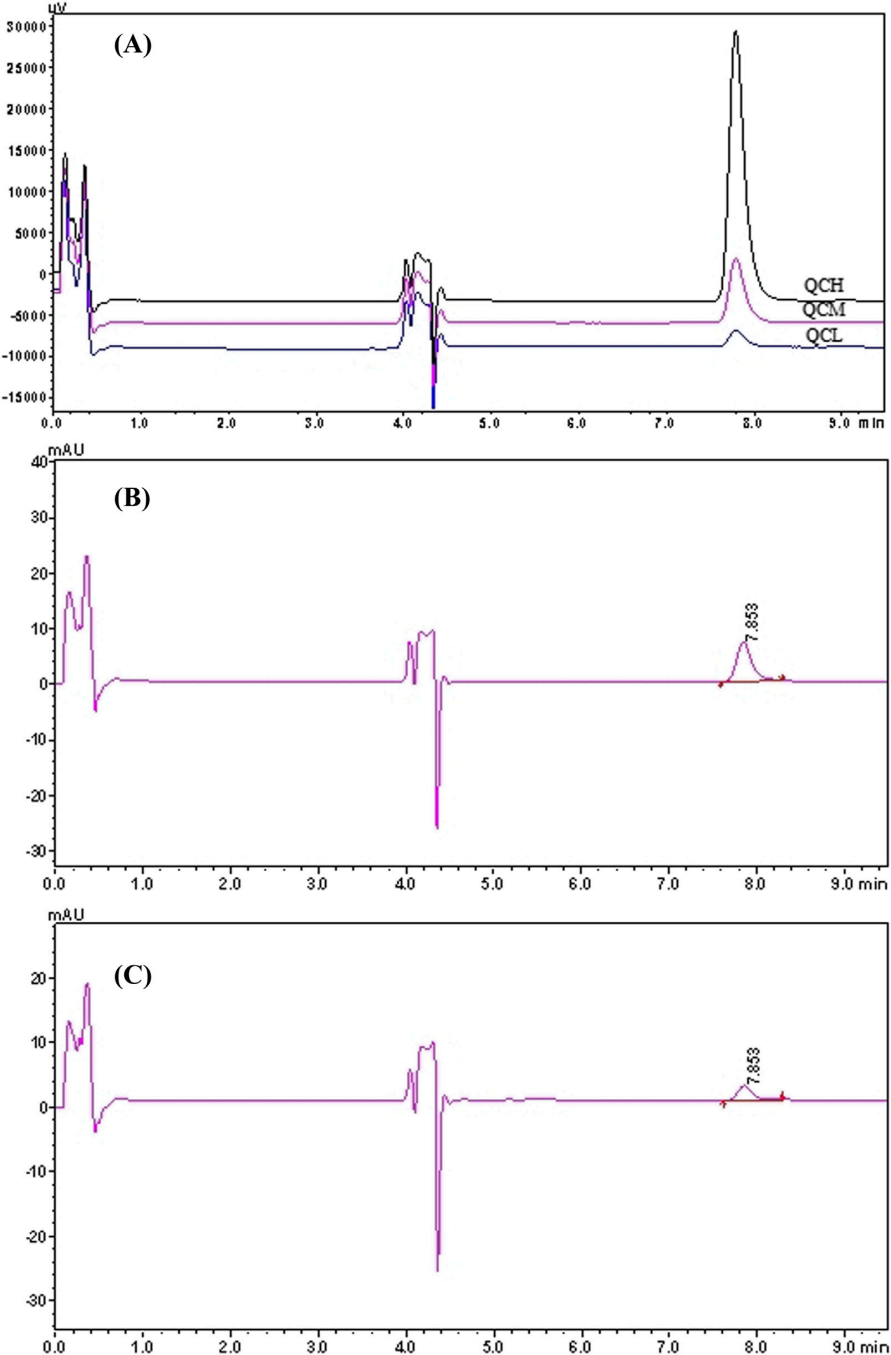
2D-LC chromatogram of Pyrotinib: QC chromatograms of pyrotinib at low, medium and high concentrations (45, 180, 720 ng/mL). **(A)** QC chromatograms of pyrotinib at medium (180 ng/mL, the retention time is 7.853 min). **(B)** Chromatograms of plasma sample from a patient administered pyrotinib (45.07 ng/mL, the retention time is 7.853 min) **(C)**.

In the 2D-LC system, pyrotinib exhibited a retention time of 7.853 min. Chromatograms of pyrotinib standards, quality control (QC) samples (QCL, QCM, and QCH), QC sample concentrations, and patient plasma samples are shown in Figures 2, 3. The chromatograms demonstrate effective separation of pyrotinib from interfering substances. The retention time consistency between patient plasma samples and QC samples confirms the specificity of the method.

### 3.2 Calibration curve and linear range

**FIGURE 4:**
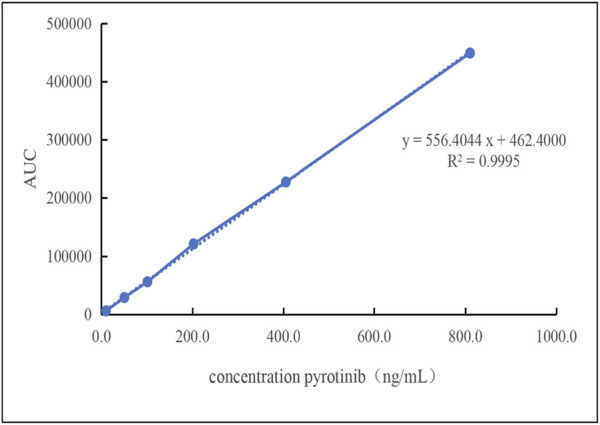
The linearity of the standard curve was observed at pyrotinib Plasma concentrations that ranged 10.10–810.40 ng/mL.

A linear regression analysis was conducted using six calibration concentrations as the independent variable (X) and the peak area as the dependent variable (Y). As shown in Figure 4, the calibration curve demonstrated excellent linearity within the range of 10.10–810.40 ng/mL, with the regression equation y = 556.4044x + 462.40 (*R* ^2^ = 0.9995). The correlation coefficient *R* ^2^ was equal to 0.9995. The lowest limit of quantitation (LLOQ) was referenced to the lowest concentration, which was 10.10 ng/mL.

### 3.3 Accuracy and precision

The results of precision and accuracy were listed in Table 3. The intra-day (RSD ≤5.30%),inter-day (RSD ≤3.80%) and accuracy (96.82%–100.60%) were within the acceptance criteria for each QC level. Both inter- and intra-day accuracy and precision values met the acceptance criteria for each QC level.

**TABLE 3:** Intra-day and inter-day precision and accuracy.

QC (nominal conc) (ng/mL)	Intra-day (n = 5)			Inter-day (n = 15)		
	Measured conc mean ± SD (ng/mL)	RSD%	Accuracy (%)	Measured conc mean ± SD (ng/mL)	RSD%	Accuracy (%)
45	45.05 ± 2.39	5.30	100.12	44.38 ± 1.69	3.80	98.62
180	174.27 ± 1.38	0.79	96.82	180.55 ± 6.15	3.41	100.31
720	712.88 ± 8.84	1.24	99.01	724.30 ± 9.28	1.28	100.60

### 3.4 Extraction recovery

The extraction recovery rates for the low, medium, and high concentrations were 100.12% ± 4.72%, 96.82% ± 1.53%, and 99.01% ± 1.10%,1.11%≤ RSD ≤4.71% (RSD≤15%) respectively (Table 4), demonstrating high and consistent recovery rates for the method.

**TABLE 4:** Extraction recovery of pyrotinib (n = 5).

Nominal conc (ng/mL)	Recovery	
	Mean ± SD (%)	RSD%
45	100.12 ± 4.72	4.71
180	96.82 ± 1.53	1.58
720	99.01 ± 1.1	1.11

Mean ± SD, mean value ± standard deviation; RSD, relative standard deviation.

### 3.5 Stability

The results indicated that QC samples of pyrotinib maintained stability under the following conditions: at RT for up to 8 h (instability was observed at 24 h), after three freeze-thaw cycles, at 4 °C for up to 48 h, and at −20 °C for up to 3 weeks. The relative standard deviations (RSD) of the measured concentrations across all conditions were less than 15% (Table 5). These results affirm the reliable stability of pyrotinib in plasma under the tested experimental conditions: 8 h at RT, three freeze-thaw cycles, 48 h at 4 °C, and 3 weeks at −20 °C.

**TABLE 5:** Stability results of pyrotinib quality control samples under different storage conditions (n = 3).

Conditions		Nominal conc (ng/mL)	Measured conc mean ± SD (ng/mL)	Accuracy (%)	RSD%
Room temperature	4 h	45	44.17 ± 1.85	97.95	4.79
		180	184.46 ± 4.07	105.43	3.11
		720	754.86 ± 16.41	105.09	2.34
	8 h	45	42.02 ± 2.08	93.23	5.93
		180	191.61 ± 26.01	95.98	3.01
		720	748.43 ± 18.24	100.47	3.46
	24 h	45	35.60 ± 0.35	78.92	0.12
		180	147.24 ± 1.37	84.15	0.50
		720	592.23 ± 10.72	82.46	2.20
Stored at 4 °C	8 h	45	52.23 ± 1.36	104.75	1.36
		180	212.54 ± 4.23	103.43	4.23
		720	864.06 ± 4.42	101.68	4.42
	24 h	45	51.48 ± 2.61	103.23	2.61
		180	215.58 ± 2.98	104.89	2.98
		720	863.65 ± 3.00	101.61	3.00
	48 h	45	57.26 ± 2.83	114.83	2.83
		180	231.1 ± 3.33	112.44	3.33
		720	932.51 ± 2.37	109.70	2.37
Stored at −20 °C	1 w	45	44.30 ± 2.20	98.30	6.00
		180	185.50 ± 5.10	106.00	2.10
		720	773.20 ± 6.20	108.20	0.60
	2 w	45	48.20 ± 0.80	106.80	2.90
		180	189.30 ± 2.20	108.20	2.00
		720	784.00 ± 4.70	109.20	1.60
	3 w	45	46.80 ± 1.40	103.80	3.70
		180	187.00 ± 5.60	106.90	2.90
		720	760.70 ± 4.40	105.90	1.50
Freeze/Thaw stability (3 cycles)		45	44.93 ± 3.74	99.62	3.74
		180	195.35 ± 10.93	105.56	10.93
		720	746.62 ± 1.37	103.93	1.37

### 3.6 Clinical application

Plasma samples were collected from HER2-positive breast cancer patients receiving pyrotinib treatment (manufactured by Jiangsu Hengrui Pharmaceuticals Co., Ltd., National Drug Approval No. H20180012; 80 mg tablets, administered once daily at doses ranging from 240 to 400 mg). Patients had taken pyrotinib for at least 7 consecutive days. The validated TDM method developed in this study was applied to measure trough concentrations (17.75–92.56 ng/mL) and peak concentrations (51.17–232.94 ng/mL) in twenty patients, respectively. The adverse reactions of patients were graded using the Common Terminology Criteria for Adverse Events (CTCAE) version 5.0. Most of the adverse reactions were diarrhea of varying degrees. Patients with higher plasma concentrations of pyrotinib experienced more severe adverse reactions, and some could not tolerate the drug, leading to dose reduction or temporary discontinuation of pyrotinib (Table 6).

**TABLE 6:** Plasma concentration measurement results of 20 cases administered different doses of pyrotinib.

No.	Age	Diagnosis	Dosage	Trough conc (ng/mL)	Peak conc (ng/mL)	Related adverse reactions
1	51	Post-left breast cancer	240 mg, po, qd	35.13	120.49	Nausea Grade 2
2	62	Post-left breast cancer	240 mg, po, qd	51.61	78.05	Diarrhea Grade 3
3	63	Post-right breast cancer	240 mg, po, qd	92.56	179.22	Diarrhea Grade 3
4	52	Post-left breast cancer	240 mg, po, qd	17.75	62.39	Diarrhea Grade 3
5	57	Post-right breast cancer	240 mg, po, qd	26.72	106.57	Anorexia Grade I
6	49	Left breast cancer	240 mg, po, qd	25.94	52.32	Diarrhea Grade 2
7	61	Right breast cancer	240 mg, po, qd	27.73	56.34	Diarrhea Grade 1
8	54	Post-left breast cancer	320 mg, po, qd	33.57	120.93	Diarrhea Grade 3
9	40	Left breast cancer	320 mg, po, qd	72.64	232.94	Diarrhea Grade 3; Hand-foot Syndrome Grade 2
10	49	Left breast cancer	320 mg, po, qd	20.16	56.87	Diarrhea Grade 2
11	34	Post-left breast cancer	320 mg, po, qd	28.37	51.17	Diarrhea Grade 1
12	34	Post-left breast cancer	320 mg, po, qd	21.51	126.77	Diarrhea Grade 2
13	53	Right breast cancer	320 mg, po, qd	18.01	104.03	Diarrhea Grade 2
14	42	Left breast cancer	320 mg, po, qd	84.28	141.99	Diarrhea Grade 2
15	52	Left breast cancer	320 mg, po, qd	59.17	104.73	Diarrhea Grade 2
16	60	Post-left breast cancer	320 mg, po, qd	23.48	120.94	Diarrhea Grade 3
17	56	Right breast cancer	400 mg, po, qd	52.94	168.84	Diarrhea Grade 2; Rash Grade 1; Pruritus Grade 1
18	57	Post-right breast cancer	400 mg, po, qd	37.98	127.07	Diarrhea Grade 2
19	49	Post-left breast cancer	400 mg, po, qd	64.01	89.13	Diarrhea Grade 1
20	38	Left breast cancer	400 mg, po, qd	43.12	78.43	Diarrhea Grade 1; Gastrointestinal bloating

## 4 Discussion

This study successfully developed and validate a method for TDM of pyrotinib in human plasma using 2D-LC system. Firstly, the clinical plasma samples of pyrotinib were collected, and then taken the supernatant after centrifugation. Finally, it can be injected into the system via the autosampler. The first-dimensional liquid chromatography column (LC1) is responsible for preconcentrating and preliminarily separating the target substance. The intermediate column is in charge of transferring the target substance to the capture column. The second-dimensional liquid chromatography column (LC2) further separates the target substance. The first-dimension column SX1 is an ion-exchange column that achieves separation based on differences in ionic charge, radius, and hydrophilicity. It offers broad compatibility and is well-suited for the analysis of various antineoplastic agents. The second-dimension column SCB is a C18 reversed-phase column that separates compounds by polarity. It provides strong retention for non-polar or weakly polar analytes, excellent selectivity, a wide pH tolerance, high stability, and a long service life. The markedly different separation mechanisms of the two columns enable efficient resolution of the complex components present in blood. The first-dimension mobile phase consisted of methanol, acetonitrile, and 65 mmol/L ammonium phosphate (in a ratio of 1:3:3, V/V/V, pH adjusted to 7.0 with triethylamine). The second-dimension mobile phase consisted of acetonitrile, isopropanol, and 10 mmol/L ammonium phosphate (in a ratio of 16:7:1, V/V/V). Because pyrotinib is a basic compound, we selected an acidic mobile phase. An acidic mobile phase can suppress the ionization of pyrotinib, thereby reducing peak tailing, improving peak symmetry, and enhancing both response and detection sensitivity. By finely tuning the flow-rate, concentration, and pH of the mobile phase, each component can be eluted in a predictable order. The online analysis of the sample can be completed at a full wavelength of 356 nm. All processes are automatically carried out under the program of the workstation. The 2D-LC workflow simplifies sample pretreatment to a single protein precipitation step and enables rapid analysis within 9.50 min, substantially improving throughput. Method validation demonstrated excellent linearity (*R* ^2^ = 0.9995) over the range of 10.10–810.40 ng/mL, with intra-day and inter-day precision (RSD) below 5.30% and 3.80%, respectively, and extraction recovery rates ranging from 96.82% to 100.12%. The experimental results all met the technical criteria for bioanalytical methods outlined in the Chinese Pharmacopoeia and the US Food and Drug Administration (FDA) (Ch.p, 2020; FDA, 2018). Stability assessments confirmed that pyrotinib in plasma remained stable under the following conditions: 8 h at room temperature (RT), three freeze-thaw cycles, 48 h at 4 °C, and 3 weeks at −20 °C. The stability results further validated the reliability of this method for real-world applications. Compared to conventional one-dimensional HPLC-UV techniques, this method significantly enhances separation efficiency and specificity through a dual-column orthogonal separation mechanism. Compared with conventional LC-MS/MS methods, the proposed 2D-LC approach offers a practical alternative for hospitals and clinical laboratories with limited access to mass spectrometry. It simplifies sample preparation, reduces operational costs, and avoids common issues such as ion suppression, making it particularly suitable for large-scale TDM programs. Owing to the accurate and reliable detection results of the 2D-LC method, as well as its simple operation and short duration, it has been widely used for the determination of plasma concentrations of various drugs (Li et al., 2021; Wang et al., 2024).

This method was validated in twenty patients with advanced HER2-positive breast cancer (dose range: 240–400 mg/day). The twenty patients were aged 34–63 years. Among them, the 40–59-year age group was the most numerous, comprising 65%. The pyrotinib exhibited significant pharmacokinetic heterogeneity, with trough and peak plasma concentrations ranging from 17.75–92.56 ng/mL and 51.17–232.94 ng/mL, respectively. Comparative analysis with previous studies revealed partial overlap in trough concentrations with the steady-state (32.60–82.80 ng/mL) reported by Zhao et al. (2021). The peak concentration range overlaps with the peak concentration data (98.70–170 ng/mL) from the 240–400 mg/day dose group in the Phase I study conducted by Ma F’s team (Ma et al., 2017).

As a HER2-targeted small-molecule tyrosine kinase inhibitor, pyrotinib exhibited dose-dependent plasma concentrations (Liu et al., 2023). Subtherapeutic concentrations may compromise efficacy, while supratherapeutic levels increase adverse reaction risks. The abnormal pharmacokinetic characteristic was observed in Patient 9 (a 40-year-old female receiving 320 mg/day) in this study. It serves as a cautionary note: her trough and peak concentrations reached 72.64 ng/mL and 232.94 ng/mL, respectively, accompanied by drug-related adverse events including Grade 3 diarrhea and Grade 2 hand-foot syndrome. She had to stop taking pyrotinib or reduce the dose of pyrotinib gradually (from 320 mg–240 mg–160 mg). Pyrotinib is primarily metabolized by CYP3A4. Concomitant use of pyrotinib with strong CYP3A4 inducers may decrease systemic exposure and potentially compromise antitumor efficacy. Concomitant use of pyrotinib with strong CYP3A4 inhibitors may increase systemic exposure and raise patient safety risks. We have checked the patient 9, She had indeed not taken any CYP3A4 inhibitors. The Patient 4 had the lowest trough concentration and a moderate peak concentration, possibly due to the concurrent use of dexamethasone, a CYP3A4 inducer, during pyrotinib treatment. She experienced Grade 3 diarrhea as an adverse reaction. During the initial phase of pyrotinib therapy, most patients develop relatively severe adverse events. However, these reactions usually diminish once tolerance is established. As can be seen from Table 6, there are large inter-individual differences in plasma concentrations among patients receiving the same dose and different doses of pyrotinib. Patients with higher plasma concentrations tend to experience more severe adverse reactions. This suggests that the pharmacokinetic profile of pyrotinib is significantly influenced by factors such as genetic polymorphisms, hepatic enzyme metabolic activity, or drug-drug interactions. Therefore, implementing TDM for pyrotinib is imperative. Through TDM, the plasma concentration of pyrotinib in patients can be monitored in real-time, ensuring that the drug concentration remains within the therapeutic window. For patients with abnormal plasma drug concentrations, the causes should be further assessed, and the treatment regimen should be adjusted accordingly, such as reducing the dose, switching medications, or combining with other therapeutic approaches. This not only reduces severe adverse reactions caused by excessively high drug concentrations and improves patient treatment adherence, but also ensures that the drug concentration stays within the therapeutic window, thereby enhancing the efficacy of pyrotinib and extending patients’ progression-free survival (PFS) and overall survival (OS). Additionally, by optimizing the treatment regimen and reducing unnecessary drug utilization and treatment interruptions, medical costs can be effectively reduced.

This study focuses on establishing and validating a 2D-LC method for pyrotinib. Due to the limited number of patients, twenty plasma samples were used to validate the reliability of the method. Comparable publications (*Biomedical Chromatography*, Li et al., 2021: n = 5; *Pharmacology*, Wang et al., 2022: n = 14) adopted similar sample sizes. We will continue to collect plasma samples from patients taking pyrotinib for further studies. In addition, We used the external standard method to determine the concentration of pyrotinib in human plasma. Although an external-standard approach was employed, the validated accuracy, precision, and recovery data confirm that the method remains reliable. We chose human plasma as the analytical matrix to develop and validate the 2D-LC method in this study. Because the whole blood is a complex matrix that can interfere with analysis and yield inaccurate results. Plasma drug concentrations reliably reflect distribution at the site of action, making them a valid surrogate for efficacy and toxicity. Moreover, plasma is also easier to collect and process - simple centrifugation separates plasma for immediate analysis, whereas whole blood requires additional steps to remove cells, increasing manipulation and error risk.

## 5 Conclusion

The validated 2D-LC assay determines pyrotinib in human plasma within 9.5 min using only protein precipitation. It offers excellent linearity, recovery and stability under varied storage conditions. Pharmacokinetic data from twenty patients (240–400 mg/day) revealed marked inter-individual variability that correlated with adverse-event severity, confirming the method’s value for individualized TDM in routine oncology practice. It’s demonstrate that the developed 2D-LC method for TDM of pyrotinib in human plasma is suitable for clinical TDM. In the future, We will explore the efficacy of pyrotinib at different plasma concentration ranges. This will help provide personalized medication recommendations for clinical settings. Our goal is to maximize therapeutic benefits and minimize adverse reactions.

## Data Availability

The original contributions presented in the study are included in the article/supplementary material, further inquiries can be directed to the corresponding authors.
